# The Human Salivary Microbiome Is Shaped by Shared Environment Rather than Genetics: Evidence from a Large Family of Closely Related Individuals

**DOI:** 10.1128/mBio.01237-17

**Published:** 2017-09-12

**Authors:** Liam Shaw, Andre L. R. Ribeiro, Adam P. Levine, Nikolas Pontikos, Francois Balloux, Anthony W. Segal, Adam P. Roberts, Andrew M. Smith

**Affiliations:** a UCL Genetics Institute, UCL, London, United Kingdom; b Centre for Mathematics and Physics in the Life Sciences and Experimental Biology (CoMPLEX), UCL, London, United Kingdom; c UCL Eastman Dental Institute, UCL, London, United Kingdom; d School of Dentistry, University Center of Pará – CESUPA, Belém, Brazil; e Division of Medicine, UCL, London, United Kingdom; f Institute of Ophthalmology, UCL, London, United Kingdom; g Moorfields Eye Hospital, London, United Kingdom; h Department of Parasitology, Liverpool School of Tropical Medicine, Liverpool, United Kingdom; University of Maryland, School of Medicine

**Keywords:** environmental microbiology, microbiome, oral microbiology

## Abstract

The human microbiome is affected by multiple factors, including the environment and host genetics. In this study, we analyzed the salivary microbiomes of an extended family of Ashkenazi Jewish individuals living in several cities and investigated associations with both shared household and host genetic similarities. We found that environmental effects dominated over genetic effects. While there was weak evidence of geographical structuring at the level of cities, we observed a large and significant effect of shared household on microbiome composition, supporting the role of the immediate shared environment in dictating the presence or absence of taxa. This effect was also seen when including adults who had grown up in the same household but moved out prior to the time of sampling, suggesting that the establishment of the salivary microbiome earlier in life may affect its long-term composition. We found weak associations between host genetic relatedness and microbiome dissimilarity when using family pedigrees as proxies for genetic similarity. However, this association disappeared when using more-accurate measures of kinship based on genome-wide genetic markers, indicating that the environment rather than host genetics is the dominant factor affecting the composition of the salivary microbiome in closely related individuals. Our results support the concept that there is a consistent core microbiome conserved across global scales but that small-scale effects due to a shared living environment significantly affect microbial community composition.

## IMPORTANCE

Previous research shows that the salivary microbiomes of relatives are more similar than those of nonrelatives, but it remains difficult to distinguish the effects of relatedness and shared household environment. Furthermore, pedigree measures may not accurately measure host genetic similarity. In this study, we include genetic relatedness based on genome-wide single nucleotide polymorphisms (SNPs) (rather than pedigree measures) and shared environment in the same analysis. We quantify the relative importance of these factors by studying the salivary microbiomes in members of a large extended Ashkenazi Jewish family living in different locations. We find that host genetics plays no significant role and that the dominant factor is the shared environment at the household level. We also find that this effect appears to persist in individuals who have moved out of the parental household, suggesting that aspects of salivary microbiome composition established during upbringing can persist over a time scale of years.

## INTRODUCTION

The human microbiome is the name given to the collected communities of bacteria that live on and in the human body. The oral microbiome is one of the most diverse (1) of any human-associated microbial community (2). The oral microbiome is a causative factor in conditions such as dental caries (3), periodontal disease (4), and halitosis (5) and has also been implicated as a reservoir for infection at other body sites (2) and in the pathogenesis of nonoral diseases, such as inflammatory bowel disease (6). Strictly speaking there is no single “oral microbiome,” as its composition is highly heterogeneous at different sites in the mouth (7, 8), but the term is commonly used to encompass all of these sites. Site-specific microbiomes can be observed in the periodontal sulcus, dental plaque, tongue, buccal mucosa, and saliva (9). The salivary microbiome exhibits long-term stability and can be considered an important reservoir that contains microorganisms from all distinct ecological niches of the oral cavity. Characterizing and understanding the factors defining the composition of the salivary microbiome are thus crucial to understanding the oral microbiome (10, 11).

Some factors that are thought to influence the human microbiome include environment, diet, disease status, and host genetics (12). The relative importance of these factors for the oral microbiome is still under debate, with the majority of previous studies focusing on the gut microbiome (7–9), although it seems reasonable to assume some potential interaction between the salivary microbiome and microbial communities in other parts of the human body, including the intestinal tract (10).

There is evidence that genetically related individuals tend to share more gut microbes than unrelated individuals do, whether or not they are living in the same house at the time of sampling (13, 14). However, the levels of covariation are similar in monozygotic and dizygotic twins, suggesting that a shared early environment may be a more important factor than genetics (13, 15). The effect of cohabitation with direct and frequent contact is greatest when considering the skin microbiome, with a less-evident effect on the gut and salivary microbiomes (11).

There is also evidence that genetic variation is linked to microbiome composition across other body sites, including the mouth (12), with a recent genome-wide association study (GWAS) identifying several human loci associated (*P* < 5 × 10^−8^) with microbial taxonomies in the gut microbiome (16). However, no study thus far has incorporated both genetic relatedness (as a continuous variable) and shared environment into the same analysis of the salivary microbiome.

Despite high diversity between individuals, the salivary microbiome appears to have little geographical structure at the genus level at the global scale (17). Nevertheless, at smaller geographical scales, it appears that the environment plays a role in the oral microbiome. Song et al. studied 60 household units and found that the bacterial composition of dorsal tongue bacterial samples was more similar between cohabiting family members than for individuals from different households, with partners and mother-child pairs having significantly more similar communities (18). However, this did not include information on genetic relatedness in addition to family relationships. It appears that household-level differences in the salivary microbiome may also apply to genetically unrelated individuals and nonpartners, with a similar pattern observed in analysis of 24 household pairs of genetically unrelated individuals, only half of whom were considered romantic couples at the time of sampling (19).

The establishment of the oral microbiome appears to proceed rapidly in the first few years of life, with a notable increase in diversity from 0 to 3 years (18), especially after the eruption of teeth (20). The plaque microbiome also appears stable within adult individuals over a period of at least 3 months, with a unique “fingerprint” of oligotypes discernible even within a single bacterial genus (21). Another study indicates that the salivary microbiome is relatively stable over a year, despite measurable effects of interventions like flossing (22). Taken together, these findings suggest the intriguing hypothesis that once a particular oral microbiome is established earlier in life, it can potentially persist for months and perhaps even years, particularly if external factors such as diet remain fixed. If this were true, shared upbringing effects would continue to be detected in the salivary microbiome even after individuals are no longer living in the same household (15).

A recently described large Ashkenazi Jewish family (23) offers an opportunity to investigate the effect of both environment and genetics in closely related individuals. The availability of host genetic data for this cohort means that we can calculate similarity between individuals based on single nucleotide polymorphisms (SNPs), rather than using measures of relatedness from pedigrees that do not precisely correspond to shared genetic content (24). We hypothesized that using this more accurate measure of host genetic similarity could lead to different conclusions about the proportion of shared microbiome composition attributable to genetics compared to previous studies. While, like other studies, we lack information on potential confounders such as diet and lifestyle (17), due to shared cultural practices between members of the ultraorthodox Ashkenazi Jewish community (25), we believe that confounding factors are likely to be more controlled for in this cohort than in others. For this reason, this cohort represents a unique opportunity to compare the salivary microbiome within a large number of individuals living in separate locations but nevertheless sharing a similar diet, lifestyle, and genetic background and to investigate the long-term effect of shared upbringing on salivary microbiome composition.

## RESULTS

### Description of cohort.

The families analyzed in this study have been already described in detail by Levine et al. (23). All individuals sampled were from the ultraorthodox Ashkenazi Jewish community. Family A comprised more than 800 individuals living in at least eight cities in four countries. Family B comprised more than 200 individuals living in at least four cities in three countries. The unrelated controls were sampled from the same community as the two families. In total, data were generated from samples from 133 individuals in family A, 18 individuals in family B, and 27 controls.

**FIG 1:**
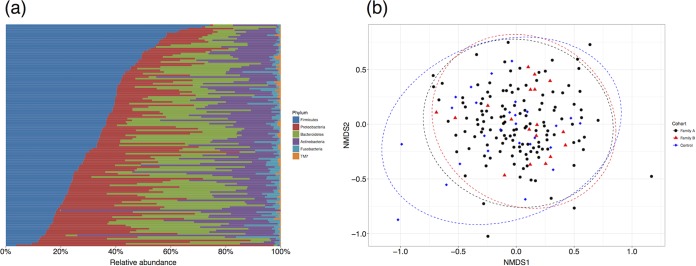
The family within this study contains a representative sample of variation in salivary microbiome composition compared to controls. (a) Relative abundance of the six bacterial phyla found in saliva samples from family A sorted by decreasing *Firmicutes* content. The color scheme used here was adapted from that in Stahringer et al. (15). The 271 MED phylotypes were assigned to taxa using RDP based on the HOMD database. (b) Nonmetric multidimensional scaling based on Bray-Curtis dissimilarity between samples shows high overlap between family A, family B, and unrelated Ashkenazi Jewish controls. NMDS1, nonmetric multidimensional scaling axis 1.

Using minimum entropy decomposition (see Materials and Methods), we found 271 phylotypes in the total microbiome data set, all of which were present when considering just family A. Of these 271 phylotypes, 49 were present in >95% of individuals within family A, with the *Firmicutes* the most abundant phyla (Fig. 1a) as observed in previous oral microbiome studies (15, 26). The most abundant genera were *Streptococcus* (30.4%), *Rothia* (18.5%), *Neisseria* (17.1%), and *Prevotella* (17.1%). The composition of samples was similar between the two families (families A and B) and the unrelated controls (Fig. 1b). These groupings had a small but significant effect in an analysis of variance (*R*^*2*^ = 0.015; *P* < 0.01), but this is typical of comparisons between such large groups that may differ in an unknown number of confounded variables (e.g., diet, genetics, lifestyle). We concluded that family A was at the very least a representative sample capturing the majority of the variation present in the wider Ashkenazi Jewish population, if not also individuals who are not Ashkenazi Jewish (for comparison with Human Microbiome Project data, see Fig. S3 in the supplemental material).

This cohort was originally collected for a study of the genetics of Crohn’s disease (23), and 28 individuals within our sample had a diagnosis of the disease at the time of saliva sample acquisition. We found no significant effect of Crohn’s disease on salivary microbiome composition with an exploratory analysis of variance (*R*^2^ = 0.009; *P* = 0.101; *n* = 148) accounting for other variables. It was therefore not included as a covariate in further analysis.

### Host genetic similarity is weakly correlated with salivary microbiome similarity.

**FIG 2:**
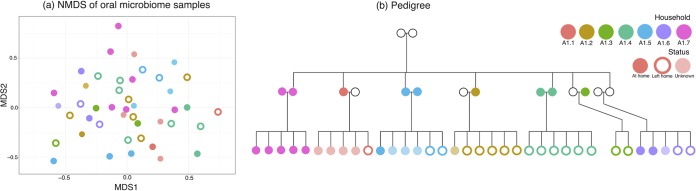
Salivary microbiome composition appears associated with household in an exploratory analysis. Salivary microbiome samples cluster somewhat by household (shown by different colors) despite large overlap between them, shown by a nonmetric multidimensional scaling based on Bray-Curtis dissimilarity between samples from individuals in a particular subfamily (*n* = 44) within family A (a). This figure includes individuals who are currently living together (filled circles), those who had moved out of their childhood home (empty circles), and those for whom data were missing (faint circles). This clustering could be due to shared environment or also due to shared genetics, as is obvious from the pedigree (b).

We performed an exploratory analysis on individuals in family A with both genetic and microbiome data available (*n* = 111) (Fig. S4) and found that genetic kinship was weakly but significantly associated with salivary microbiome dissimilarity computed using Bray-Curtis dissimilarity (Fig. S5a; r = 0.065 by Mantel test; *P* = 0.001). This analysis does not take into account confounding by the shared environment and therefore sets a probable upper bound on the variation that can be attributed to host genetics. An exploratory analysis of microbiome variation across a subfamily within family A (*n* = 44) showed that individuals from the same household had a more similar microbiome composition as measured by Bray-Curtis dissimilarity (mean ± standard deviation [SD], 0.623 ± 0.088) compared with individuals from different households (0.652 ± 0.084), and this difference was significant (*P* < 0.001 by two-sided *t* test). An exploratory visual representation of this variation showed further clustering by household, although there was large overlap between households in two dimensions (Fig. 2). However, such an analysis is insufficient; household is obviously correlated with variation in host genetics (Fig. S6) because parents tend to live with their children. This emphasizes the need for a quantitative approach looking at the effect of both household and genetics simultaneously as well as other potential confounders.

The approach we chose to use was adonis, which performs a permutational analysis of variance in community composition using a sequential sum-of-squares approach (27). We used Bray-Curtis dissimilarities to quantify differences in salivary microbiome composition between individuals. The following sections present our analyses attempting to quantify the effects of shared environment and genetics. The analysis groups were effectively nested as follows: individuals cohabiting with at least one other individual (*n* = 26; Table 1), individuals who had cohabited with at least one other individual, either at the time of sampling or beforehand (*n* = 61; Table 2), individuals living in four different cities who were not necessarily cohabiting with another individual (*n* = 82; Table S1), and individuals with host genetic information available (*n* = 111; Table 3).

10.1128/mBio.01237-17.9 TABLE S1  Permutational analysis of variance for 82 individuals who lived in four different cities, showing that city has no significant effect on salivary microbiome composition. Individuals were not necessarily cohabiting with others. Download TABLE S1, DOCX file, 0.05 MB. Copyright © 2017 Shaw et al. 2017 Shaw et al. This content is distributed under the terms of the Creative Commons Attribution 4.0 International license.

**TABLE 1:** Permutational analysis of variance (adonis) results for 26 cohabiting individuals who lived in the same household with at least one other individual^a^

Variable^b^	City only		Household only		City and household^c^	
	*R*^2^	*P*	*R*^2^	*P*	*R*^2^	*P*
Sequencing plate	0.048	0.190	0.048	0.075	0.048	0.458
Gender	0.032	0.724	0.032	0.4	0.032	0.467
Age	0.069	0.017	0.069	0.004	0.069	0.013
MDS1	0.031	0.757	0.031	0.537	0.031	0.727
MDS2	0.050	0.142	0.050	0.052	0.050	0.099
MDS3	0.030	0.807	0.030	0.585	0.030	0.862
MDS4	0.049	0.162	0.049	0.054	0.049	0.097
MDS5	0.029	0.824	0.029	0.614	0.029	0.791
City	0.080	0.400			0.080	0.178
Household			0.300	0.001	0.220	0.001
Residuals	0.582		0.362		0.362	
Total	1.000		1.000		1.000	

a Sharing a household was always significant and explained the most variance of any variable (>18%) even in a model that nests permutations within cities.

b The order of variables in the model is given by their order in the table. MDS1 to MDS5 are the five axes of metric multidimensional scaling (MDS).

c Permutations stratified by city in this analysis.

**TABLE 2:** Permutational analysis of variance (adonis) results for 61 individuals who had at least cohabited at some point^a^

Variable^b^	City only		Household only		City and household^c^	
	*R*^2^	*P*	*R*^2^	*P*	*R*^2^	*P*
Sequencing plate	0.029	0.018	0.029	0.012	0.029	0.013
Gender	0.018	0.258	0.018	0.219	0.018	0.257
Age	0.038	0.002	0.038	0.001	0.038	0.002
MDS1	0.014	0.668	0.014	0.607	0.014	0.740
MDS2	0.017	0.362	0.017	0.305	0.017	0.440
MDS3	0.020	0.173	0.020	0.141	0.020	0.263
MDS4	0.020	0.150	0.020	0.118	0.020	0.147
MDS5	0.012	0.783	0.012	0.744	0.012	0.943
City	0.056	0.149			0.056	0.934
Household			0.239	0.021	0.183	0.044
Residuals	0.777		0.594		0.594	
Total	1.000		1.000		1.000	

a Sharing a household was always significant and explains the most variance of any variable (>18%) even in a model that nests permutations within cities.

b The order of variables in the model is given by their order in the table.

c Permutations stratified by city in this analysis.

**TABLE 3:** Comparison of pedigree-based and genome-wide measures of kinship to take host genetics into account in a permutational analysis of variance (adonis) on salivary microbiome dissimilarities of 111 individuals^a^

Variable^b^	Pedigree (kinship2)		Genome-wide SNPs (LDAK)	
	*R*^2^	*P*	*R*^2^	*P*
Sequencing plate	0.028	<0.001	0.028	<0.001
Gender	0.011	0.094	0.011	0.096
Age	0.023	<0.001	0.023	<0.001
MDS1	0.010	0.174	0.011	0.119
MDS2	0.007	0.706	0.010	0.231
MDS3	0.012	0.063	0.011	0.131
MDS4	0.016	0.009	0.011	0.111
MDS5	0.009	0.325	0.007	0.617
Parental household	0.215	<0.001	0.217	<0.001
Residuals	0.670		0.671	
Total	1		1	

a Using pedigree information to produce kinship results in a significant association with human genetics via the fourth MDS axis, which is not present using kinships calculated with LDAK based on genome-wide SNPs.

b The order of variables in the model is given by their order in the table.

### Shared household is the dominant factor affecting salivary microbiome composition.

We performed a permutational analysis of variance on the salivary microbiome dissimilarities for 26 individuals within family A, each of whom lived in a household with at least one other individual in the cohort. At the time of sampling, these cohabiting individuals lived in a total of 16 households in four cities (cities I, II, III, and IV). To account for host genetics, we included axes from a metric multidimensional scaling (MDS) of pairwise genetic distances between individuals as explanatory variables (see Materials and Methods and Fig. S6). The magnitude of the effect of a significant variable is given by the amount of variance explained (*R*^2^ in tables).

There was no significant effect of any of the MDS axes, suggesting that host genetics in closely related individuals does not significantly affect microbiome composition. We investigated the effect of environment using two levels of geography: city and household (Table 1). A city-only model showed no significant effect of environment (*R*^2^ = 0.08; *P* = 0.4), whereas a household-only model showed a significant effect (*R*^2^ = 0.30; *P* = 0.001). This was reproduced in a model containing both geographical variables, with permutations stratified by city, where household was still a significant effect (*R*^2^ = 0.22; *P* = 0.001), suggesting that differences at the level of household are more important than at larger geographical scales. We confirmed that city-level effects were small by extending our sample to 82 individuals across the four cities who were not necessarily cohabiting with others (48 individuals for city I, 13 for city II, 12 for city III, and 9 for city IV) and found that city still had a small effect, although it was significant (*R*^*2*^ = 0.053; *P* < 0.01). In this analysis, we also found no significant effect of genetics, but age was significant (*R*^*2*^ = 0.028; *P* = 0.0101) (Table S1).

### Spouses share taxa at the subgenus level.

**FIG 3:**
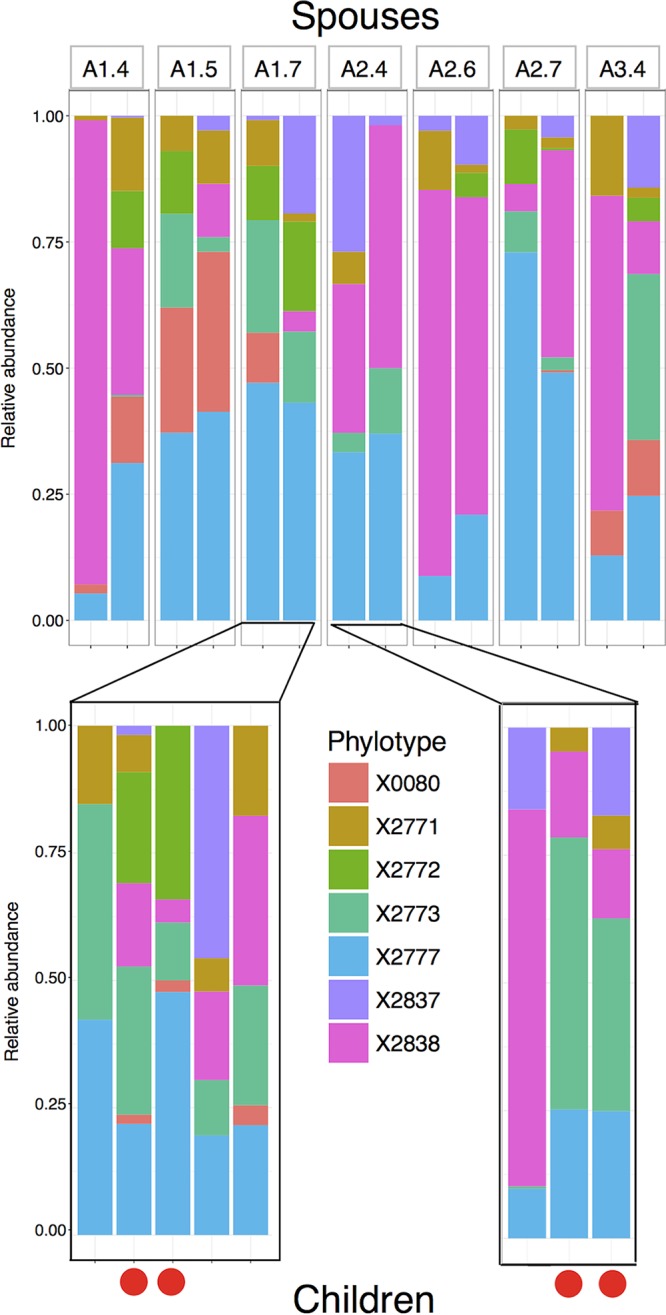
Variation within a genus shows household-level differences in relative abundances of phylotypes, shown here with the relative abundance of phylotypes within *Leptotrichia*. The relative abundance of phylotypes within seven pairs of spouses shows clear associations with household, with spouses significantly more similar in phylotype composition within *Leptotrichia* (*P* = 0.039 by two-sided *t* test). These patterns are to some extent recapitulated in their children. Looking at children still living at home, MED phylotype X2772 is not observed in any individual from household A2.4 but is found in both spouses and two children living in household A1.7. Solid red circles indicate children ≤10 years at the time of sampling, who appear more similar to each other than other pairs of children. For variation within the top 12 most abundant genera between spouses, see Fig. S7 in the supplemental material.

Restricting the analysis to only married couples within family A (*n* = 16; eight couples), shared household explained even more of the variance (*R*^*2*^ = 0.591; *P* = 0.001). Subtle variations in the relative abundance of phylotypes within the same genus between households were observable, even within the same city location. For example, *Leptotrichia* phylotypes qualitatively varied consistently between spouse pairs, and these patterns were also seen in children living at home (Fig. 3). Minimum entropy decomposition (MED) phylotype X2772 was present in both spouses in household A1.7 and was also present in the two youngest children within that household (≤10 years old). Similarly, within household A2.4, the *Leptotrichia* phylotypes of the two children who were ≤10 years old were more similar than the *Leptotrichia* phylotype of an older child. Quantitatively, repeating the permutational analysis of variance based only on the composition of phylotypes within *Leptotrichia* showed that spousal pair explained 68.4% of variance, although this was not significant (*R*^*2*^ = 0.684; *P* = 0.068), and spouses had significantly more similar subgenus phylotype composition than nonspouses did (Fig. S7b). Similar patterns with spouses were also visible in other abundant genera (Fig. S7a), with spouses on average having a significantly more similar subgenus phylotype composition than nonspouses did (mean ± standard error [SE] difference in Bray-Curtis dissimilarity for each genus, −0.048 ± 0.013; Fig. S7b).

### Household effects persist in individuals who are no longer cohabiting.

There were an additional 35 individuals who had grown up in a household with at least one other individual present but who no longer lived together at the time of sampling. To see whether the effects of household persisted, we repeated analysis of variance with these individuals included along with the cohabiting individuals (*n* = 61; Table 2). The effect of household remained significant (*R*^*2*^ = 0.183; *P* = 0.044), and no axes of human genetic variation were significant (*P* > 0.05). Age had a significant effect (*R*^*2*^ = 0.038; *P* < 0.01).

Other variables such as age and sequencing plate had smaller effects than household in all our analyses of variance. However, we chose the order of variables as presented in our tables (Tables 1, 2, and 3) to test for the effect of household after controlling for other variables. Because of the sequential sum-of-squares approach used by adonis, this ordering of variables can have an effect with an unbalanced design. To check that this was not biasing our results and therefore our conclusions about the important factors for salivary microbiome composition, we also investigated the effect of randomly permuting the order of variables in our model formula (see supplemental material). The results of this analysis confirmed that household was always significant (false-discovery rate [*q*] < 0.05, with Benjamini-Hochberg multiple testing correction), as was age.

### Relying on pedigree kinships produces a genetic signal.

To test whether our conclusions required using kinships estimated from genome-wide SNP data for individuals or whether pedigree information was sufficient, we also repeated our analyses using pedigree kinships (see Materials and Methods). Using pedigree kinships resulted in a small but significant amount of variation in microbiome composition being attributable to host genetics via MDS axis 4 (*R*^*2*^ = 0.016; *P* < 0.01; Table 3).

## DISCUSSION

We have conducted, to our knowledge, the first simultaneous investigation of the role of environment and host genetics in shaping the human salivary microbiome in a cohort of closely related individuals within a large Ashkenazi Jewish family. We found a weak correlation between host kinship and salivary microbiome dissimilarity before taking shared household into account and an apparent small but significant effect of genetics when using kinships based on the family pedigree as proxies for genetic similarity. However, when using kinship estimates based on genome-wide SNPs between individuals and simultaneously controlling for shared household with a permutational analysis of variance, we find no support for any clear effect of human genetics, suggesting that shared environment has a much larger effect than genetics and is the dominant factor affecting the salivary microbiome. Typically shared household had an order of magnitude greater effect compared with other significant variables. For example, in our analysis where city was also used as an environmental variable, the variance explained was as follows: household (18.3%), age (3.8%), and sequencing plate (2.9%) (Table 2).

We also observed that younger children living in the same household shared subtle variations in phylotype abundance within genera with their parents (Fig. 3). However, despite a persistence of household effects, it would be wrong to conclude that the salivary microbiome is completely fixed once established, as it clearly has aspects that can change over time. For example, shared household explained more variation for spousal pairs (likely due to frequent contact between them) and that phylotypes observed in younger children and their parents were not seen in older children (likely due to less frequent contact between them). Taken together, these observations support the view that human genetics does not play a major role in shaping the salivary microbiome, at least not in individuals of the same ethnicity, compared to the environment and contact with other individuals.

Our results confirm the seemingly paradoxical situation that the salivary microbiome is largely consistent across global geographical scales but can show large variation between households in the same city. Previous studies have also found evidence of small variations in salivary microbiome composition comparing samples across a global scale (17). As noted previously, this variation could be influenced by differences in environmental or cultural factors, in which case controlling for these differences would decrease the amount of geographical variation. All individuals in our study follow a traditional Ashkenazi Jewish lifestyle and subsequently are thought to share a similar diet and lifestyle regardless of geographical location (25), which may reduce the variation attributable to city-level differences.

The establishment of the oral microbiome early in life may lead to the persistence of a similar composition over several years. The microbial composition of sites within the mouth has been previously observed to be persistent within individuals over periods of months (21) to a year (22), and we see similar strain-level variation between spouses and their young children as observed between individuals by Utter et al. (21) (Fig. 3). Our results indicate the persistence of household effects in individuals no longer cohabiting, suggesting that the salivary microbiome composition established early in life via shared upbringing is able to persist for at least several years. It has been observed that monozygotic twins do not have significantly more similar gut microbiomes than dizygotic twins (13). Stahringer et al. observed the same effect in the salivary microbiome and also found that the salivary microbiomes of twins became less similar as they grew older and ceased cohabiting, concluding that “nurture trumps nature” in the salivary microbiome (15). Our findings from a large number of related individuals rather than twins support this view, including the persistence of shared upbringing effects. Shared upbringing appears to be the dominant factor affecting microbiome composition in both the gut and the mouth, rather than genetic similarity. This may have implications for understanding the familial aggregation of diseases such as inflammatory bowel disease, which has been suggested to have an environmental component (28).

The salivary microbiome appears to be far more resilient to perturbation than the gut microbiome is, with a rapid return to baseline composition after a short course of antibiotics (29). While this could be because of the pharmacokinetics of the antibiotics involved, Zaura et al. speculate that this difference may be due to the salivary microbial ecosystem’s higher intrinsic resilience to stress, as the mouth is subject to more frequent perturbation (30). Our work supporting the dominant role of the environment in affecting salivary microbiome composition suggests that another important factor in long-term persistence may be the regular reseeding of the ecosystem with bacteria from the external environment.

The fact that we reached our conclusion about the lack of effect of genetics only after including kinship based on genome-wide SNP markers casts doubt on the reliability of pedigrees for calculating relatedness. There are several possible reasons for a discrepancy between kinship estimates from pedigrees and allele sharing (24). One possibility is errors in the pedigree, most likely due to extrapair paternities, although this explanation can be ruled out in this data set. More importantly, inherent stochasticity in the Mendelian process of inheritance means that although parents always pass on 50% of their genes to their offspring, SNPs are inherited together in blocks (i.e., haplotypes), meaning that the relatedness between two offspring in a family can be substantially different from 50%. Finally, and most importantly for this closely related population, shallow pedigrees cannot fully capture complex inbreeding patterns. Thus, while pedigrees are a good model for host relatedness in microbiome studies of large randomly mating populations, they should be used with caution in closely related large families like this one.

### Limitations.

Because all individuals in our main cohort were members of the same extended Ashkenazi Jewish family, the genetic variation in our data set is therefore much lower than between individuals from a wider population. It is conceivable that host genetics between more distantly related individuals may play a significant role in affecting salivary microbiome composition. However, we note that a recent study of the nasopharyngeal microbiome among Hutterite individuals (a founder population in North America) detected associations between host variation and microbial composition with a similar cohort size (31), demonstrating that limited genetic variation can be associated with the composition of other microbiomes; it may simply be that the salivary microbiome is relatively unaffected by such variation.

Furthermore, our study looked at only overall genetic similarity, assessed using community comparison metrics based on taxon abundances. They therefore do not preclude the existence of fine-scale links between particular microbial taxa and individual genetic loci, particularly in immune-sensing genes such as those identified in the gut microbiome by Bonder et al. using a much larger cohort (32), although our study was not designed or have the statistical power to detect such associations.

Additionally, we lack detailed information on diet and lifestyle factors of individuals in this study. However, the shared cultural practices within this ultraorthodox Ashkenazi Jewish family mean that it is not unreasonable to assume that they share similar lifestyles and diet despite living in different locations around the world (25).

The apparent persistence of shared upbringing could be confounded by the fact that individuals may continue living near the household where they grew up. If this were the case, then our observation could instead be due to the persistence of a shared environment beyond the household at a level intermediate between household and city, rather than the persistence of a stable salivary microbiome following environmental change. Finally, our samples represent only a single cross-sectional snapshot in time. More long-term longitudinal studies like the work of Stahringer et al. on twins (15) are necessary to investigate the persistence of the salivary microbiome after its establishment early in life in a variety of relatedness settings.

### Conclusion.

In summary, our results incorporating a measure of genetic relatedness using SNPs demonstrate that the overall composition of the human salivary microbiome in a large Ashkenazi Jewish family is largely influenced by shared environment rather than host genetics. An apparent significant effect of host genetics using pedigree-based estimates disappears when using genetic markers instead, which shows that in future microbiome research, the use of pedigree relatedness as a proxy for host genetic similarity should be done with caution. Geographical structuring occurs to a greater extent at the household level within cities than between cities on different continents. Living in the same household is associated with a more similar salivary microbiome, and this effect persists after individuals have left the household. This is consistent with the long-term persistence of the salivary microbiome composition established earlier in life due to shared upbringing.

## MATERIALS AND METHODS

### Ethics.

Ethical and research governance approval was provided by the National Research Ethics Service London Surrey Borders Committee and the UCL Research Ethics Committee. Written informed consent was provided by all participants.

### Cohort.

Our cohort contained data from 133 individuals within the same extended family (family A) living in four disparate cities (I, II, III, and IV) across three continents (see reference 23 for more information). We also had samples available from 18 individuals from a separate smaller family (family B) and 27 unrelated Ashkenazi Jewish controls. All individuals studied were of genetically confirmed Ashkenazi Jewish ancestry (23, 25). When information was not directly available, shared household was inferred according to age; individuals within this community marry and subsequently leave the family home at a median age of 21 years (95% confidence interval, 19 to 26 years) (25). Therefore, we assumed that individuals aged 18 or younger at the time of sampling were living with their parents and individuals aged 25 or older were not.

For analysis of the effects of household, we included only households with two or more individuals so as to remove the possibility that we were measuring only interindividual differences, which can be large in the salivary microbiome (17, 21). Twenty-six individuals were living with at least one other individual at the time of sampling in a total of nine households. An additional 35 individuals who had grown up in a shared household with at least one other individual in the cohort but who were no longer living together were subsequently included in the analysis.

### Sampling.

Saliva samples were collected in sterile tubes containing saliva preservative buffer by the method of Quinque et al. (33). For the full protocol, see the supplemental material. Five hundred milliliters of saliva/preservative buffer was used with PurElute bacterial genomic kit (Edge Biosystems, Gaithersburg, MD) for DNA extraction. After bacterial DNA extraction, three spike DNAs were added to all samples at a final concentration of 4 pg/ml, 0.4 pg/ml, and 0.08 pg/ml, respectively.

### PCR amplification, purification, and sequencing.

The Mastermix 16S basic PCR kit containing MolTaq 16S DNA polymerase (Molzym GmbH & Co. KG, Bremen, Germany) was used to generate PCR amplicons. PCR amplicons were purified in two rounds using the Agencourt AMPure system (Beckman Coulter, Inc., Beverly, MA) in a Hamilton StarLet (Hamilton Company, Boston, MA) automated liquid handler. DNA quantitation and quality control were performed using the Agilent 2100 Bioanalyzer system (Agilent Technologies, Santa Clara, CA).

We used 785F (F stands for forward) and 1175R (R stands for reverse) 16S rRNA primers (see Text S1 in the supplemental material) that amplified the V5-V7 region of the 16S rRNA gene on the Illumina MiSeq system (Illumina, San Diego, CA).

### Quality control.

To assess technical variation across runs, we spiked samples during library preparation with a fixed amount of synthetic DNA (see supplemental material). Three unique spike sequences (length of 350 bases) that could be easily identified for quality control purposes were designed. We found, as expected, that the number of spike sequences and the number of putative 16S sequences (length between 350 and 380 bases) were negatively correlated with each other due to the limited total sequencing depth of the Illumina MiSeq system (Fig. S1a). The variation in reads corresponding to this spike across samples was independent of run. We also resequenced a subset of samples without spikes to verify whether spikes affected our analyses and observed the same qualitative differences (Fig. S1b), implying that the addition of spikes did not have a negative impact on downstream analysis. Paired-end reads were merged with fastq-mergepairs in VSEARCH v1.11.1 (34), discarding reads with an expected error of >1. As the expected length of the V5-V7 region was 369 bases, we discarded sequences with <350 or >380 bases.

10.1128/mBio.01237-17.2 FIG S1  Synthetic DNA spikes added during library preparation do not have an important effect on our analysis. (a) Number of reads from synthetic DNA spikes of fixed concentrations added to samples during library preparation was weakly negatively correlated with the number of reads corresponding to true 16S reads. (b) Duplicated samples with and without spikes added during library preparation showed the same qualitative differences between households, indicating that the addition of spikes did not negatively affect downstream analysis. Download FIG S1, TIF file, 11.8 MB. Copyright © 2017 Shaw et al. 2017 Shaw et al. This content is distributed under the terms of the Creative Commons Attribution 4.0 International license.

### Clustering and taxonomic classification.

Sequences were clustered with minimum entropy decomposition (MED) (35). MED requires that the variation in read depth across samples does not differ by several orders of magnitude, so we discarded samples with fewer than 5,000 reads and subsampled to a maximum number of 20,000 sequences, resulting in 6,353,210 sequences. We ran MED v2.1 with default parameters (see Text S1 in the supplemental material), identifying 271 phylotypes in the data set (Table S2). MED offers higher resolution than operational taxonomic unit (OTU) picking methods do, and it has previously been shown to differentiate the composition of the oral microbiomes of individuals over time even within the same genus in a study of plaque samples (21). We verified that using MED phylotypes gave very similar compositional dissimilarities compared to using *de novo* OTUs clustered at 98.5% sequence similarity (Fig. S2) but allowed slightly increased statistical power in analysis of variance (see supplemental material), consistent with the literature (35). MED phylotypes had taxonomy assigned using RDP (36) against the Human Oral Microbiome Database (HOMD) (37). Comparison to Human Microbiome Project (HMP) samples from various sites in the mouth also indicated that Ashkenazi Jewish individuals do not have a significantly different oral microbiome from those of other populations, with Ashkenazi Jewish saliva samples clustering with nonplaque samples from individuals in the HMP (Fig. S3). However, the use of different primers makes it difficult to reach a robust conclusion on this point.

10.1128/mBio.01237-17.1 TEXT S1  Expanded protocol, detailed methods, and details of statistical analysis Download TEXT S1, DOCX file, 0.04 MB. Copyright © 2017 Shaw et al. 2017 Shaw et al. This content is distributed under the terms of the Creative Commons Attribution 4.0 International license.

10.1128/mBio.01237-17.3 FIG S2  MED and OTU picking gave similar results. Comparison of Bray-Curtis dissimilarity between samples calculated using compositions from minimum entropy composition (MED) and operational taxonomic units (OTUs) shows a high correlation between methods (Spearman’sρ = 0.88; *P* < 0.001). This correlation is expected, as both methods should identify sequences similarly to the genus level. Download FIG S2, TIF file, 11.3 MB. Copyright © 2017 Shaw et al. 2017 Shaw et al. This content is distributed under the terms of the Creative Commons Attribution 4.0 International license.

10.1128/mBio.01237-17.4 FIG S3  NMDS ordination of samples from this study (“ashkenazi”) compared to samples from the Human Microbiome Project (HMP). Ashkenazi salivary microbiome samples were more similar to saliva microbiome samples and microbiome samples from other nonplaque sites in the human mouth. Ashkenazi samples group near but separately from the HMP saliva samples, but we cannot distinguish whether this is due to a batch effect or a real biological difference. Site abbreviations: BM, buccal mucosa; HP, hard palate; KG, keratinized gingivae; PT, palatine tonsils; SUBP, subgingival plaque; SUPP, supragingival plaque; SV, saliva; TD, tongue dorsum; TH, throat. Download FIG S3, TIF file, 13.1 MB. Copyright © 2017 Shaw et al. 2017 Shaw et al. This content is distributed under the terms of the Creative Commons Attribution 4.0 International license.

10.1128/mBio.01237-17.5 FIG S4  Pedigree for individuals in family A. The diamonds are filled on the left for ≤18 years old and filled on the right for ≥25 years old. Unsampled individuals are indicated by gray diamonds. There are three main branches of the family, which can also be seen in multidimensional scaling of the genetic distances between individuals (Fig. S6). Download FIG S4, TIF file, 41.5 MB. Copyright © 2017 Shaw et al. 2017 Shaw et al. This content is distributed under the terms of the Creative Commons Attribution 4.0 International license.

10.1128/mBio.01237-17.10 TABLE S2  Abundances per sample of MED phylotypes identified in the data set, along with their taxonomic classification and representative sequences. This table is an Excel spreadsheet. Download TABLE S2, XLSX file, 0.3 MB. Copyright © 2017 Shaw et al. 2017 Shaw et al. This content is distributed under the terms of the Creative Commons Attribution 4.0 International license.

### Inclusion of host genetics.

We investigated the effect of relatedness between individuals on salivary microbiome composition using both genetic kinships (based on SNPs) and pedigree kinships (based on the pedigree). We calculated pedigree kinships with kinship2 (38) and genetic kinships with LDAK v5.94 (39) using genome-wide SNP data from either the Illumina HumanCytoSNPv12 (Illumina, USA) or the Illumina HumanCoreExome-24, as described previously (23). These genetic kinships *k*_*g*_ are normalized to have a mean of zero and correspond to genetic similarity between individuals. *k*_*g*_ correlates with the pedigree kinship *k*_*p*_, but there can be substantial spread around the expected values due to the random nature of genetic inheritance (Fig. S5b), making *k*_*g*_ a more accurate measure of true genetic similarity between individuals (24). We converted these kinships to dissimilarities and then Euclidean distances (supplemental material) which were used in a multidimensional scaling (MDS) ordination (Fig. S6). Following Blekhman et al. (12), we used MDS with five axes as covariates in a permutational analysis of variance of salivary microbiome dissimilarities.

10.1128/mBio.01237-17.6 FIG S5  Genetic dissimilarities based on SNPs weakly correlate with salivary microbiome dissimilarities and are different from pedigree kinships. (a) Pairwise genetic dissimilarity appears weakly but significantly correlated with pairwise salivary microbiome dissimilarity (Mantel statistic *r* = 0.065; *P* = 0.001). Genetic dissimilarity was based on kinship calculated with LDAK, such that higher values indicate lower relatedness. Salivary microbiome dissimilarity was calculated with the Bray-Curtis metric. The rough clusters visible from left to right correspond to siblings-parent-child pairs (A), first cousins (B), and less-related individuals (C). (b) Correlation of observed calculated kinship with LDAK against the expected kinship from the pedigree, showing pairwise kinship estimates before rescaling to dissimilarities (see above). (c) Pairwise Euclidean distances calculated from rescaled dissimilarities. Download FIG S5, TIF file, 14.9 MB. Copyright © 2017 Shaw et al. 2017 Shaw et al. This content is distributed under the terms of the Creative Commons Attribution 4.0 International license.

10.1128/mBio.01237-17.7 FIG S6  Multidimensional scaling of kinships shows the familial structure of the cohort. This example of multidimensional scaling of samples based on kinships was calculated using LDAK showing MDS axes 1 and 2. The family structure is visible (colors) from the three arms of the pedigree (Fig. S4). The first five MDS axes were used to describe host genetic variation, following Blekhman et al. (12). Stress = 0.06. Download FIG S6, TIF file, 5.9 MB. Copyright © 2017 Shaw et al. 2017 Shaw et al. This content is distributed under the terms of the Creative Commons Attribution 4.0 International license.

10.1128/mBio.01237-17.8 FIG S7  Spouse pairs significantly share taxa at the subgenus level. (a) Relative abundance of phylotypes within the top 12 most abundant genera for 7 spouse pairs show small-scale environmental effects at the level of household. (b) The difference in mean Bray-Curtis dissimilarity (shown in red, with 95% confidence intervals in black) for spouses versus nonspouses at the subgenus level, i.e., calculated using only the phylotypes within that genus. The similarity at subgenus level is significantly lower for spouses on average across these genera, as indicated by the red line. Download FIG S7, TIF file, 14 MB. Copyright © 2017 Shaw et al. 2017 Shaw et al. This content is distributed under the terms of the Creative Commons Attribution 4.0 International license.

### Statistical analysis.

We calculated Bray-Curtis dissimilarities between samples based on relative abundances of phylotypes, excluding samples with fewer than 1,000 reads. Variance explained in Bray-Curtis dissimilarities was calculated using the adonis function from the vegan v2.4.1 package in R, which performs a permutational analysis of variance of distance matrices (27). We used 9,999 permutations, with permutations stratified by geographical sample location where appropriate.
