# Supplementary material for: The Human Salivary Microbiome Is Shaped by Shared Environment Rather than Genetics: Evidence from a Large Family of Closely Related Individuals
Source: mBio. 2017 Sep 12;8(5):e01237-17. doi: 10.1128/mBio.01237-17 (PMC5596345; doi:10.1128/mBio.01237-17)
Supplement: TABLE S1 [file mbo004173481st1.docx]

|  | **Df** | **SumsOfSqs** | **MeanSqs** | **F.Model** | **R2** | **Pr(>F)** |
| --- | --- | --- | --- | --- | --- | --- |
| Sequencing plate | 1.000 | 0.493 | 0.493 | 2.401 | 0.028 | 0.001 |
| Gender | 1.000 | 0.266 | 0.266 | 1.296 | 0.015 | 0.136 |
| Age | 1.000 | 0.499 | 0.499 | 2.430 | 0.028 | 0.001 |
| MDS1 | 1.000 | 0.163 | 0.163 | 0.794 | 0.009 | 0.775 |
| MDS2 | 1.000 | 0.224 | 0.224 | 1.092 | 0.013 | 0.311 |
| MDS3 | 1.000 | 0.262 | 0.262 | 1.278 | 0.015 | 0.141 |
| MDS4 | 1.000 | 0.309 | 0.309 | 1.507 | 0.017 | 0.050 |
| MDS5 | 1.000 | 0.225 | 0.225 | 1.095 | 0.013 | 0.306 |
| City | 3.000 | 0.942 | 0.314 | 1.530 | 0.053 | 0.005 |
| Residuals | 70.000 | 14.368 | 0.205 |  | 0.809 |  |
| Total | 81.000 | 17.751 |  |  | 1.000 |  |
