# Supplementary figures and images for: The Human Salivary Microbiome Is Shaped by Shared Environment Rather than Genetics: Evidence from a Large Family of Closely Related Individuals
Source: mBio. 2017 Sep 12;8(5):e01237-17. doi: 10.1128/mBio.01237-17 (PMC5596345; doi:10.1128/mBio.01237-17)

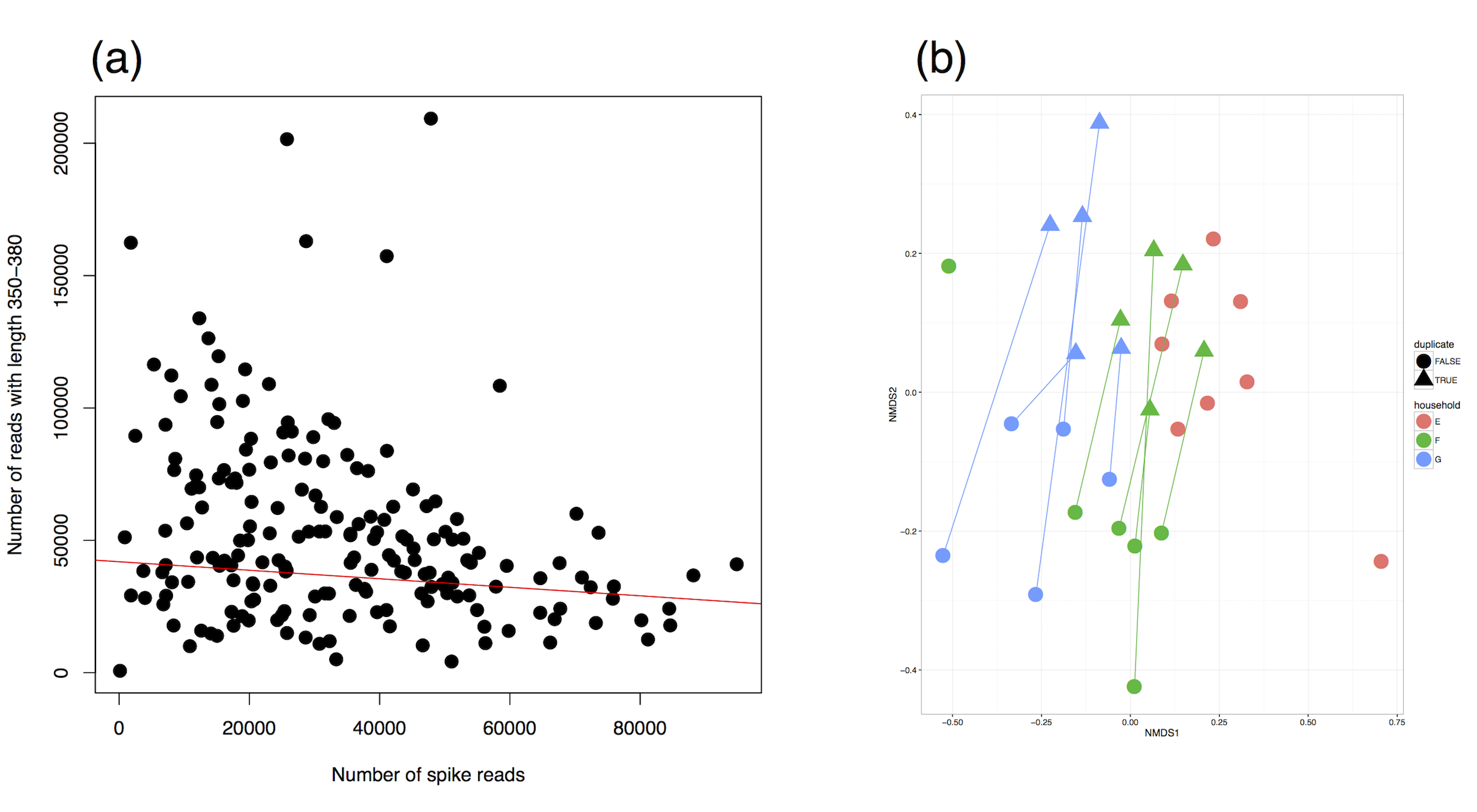

Supplement: FIG S1 [file mbo004173481sf1.tif]

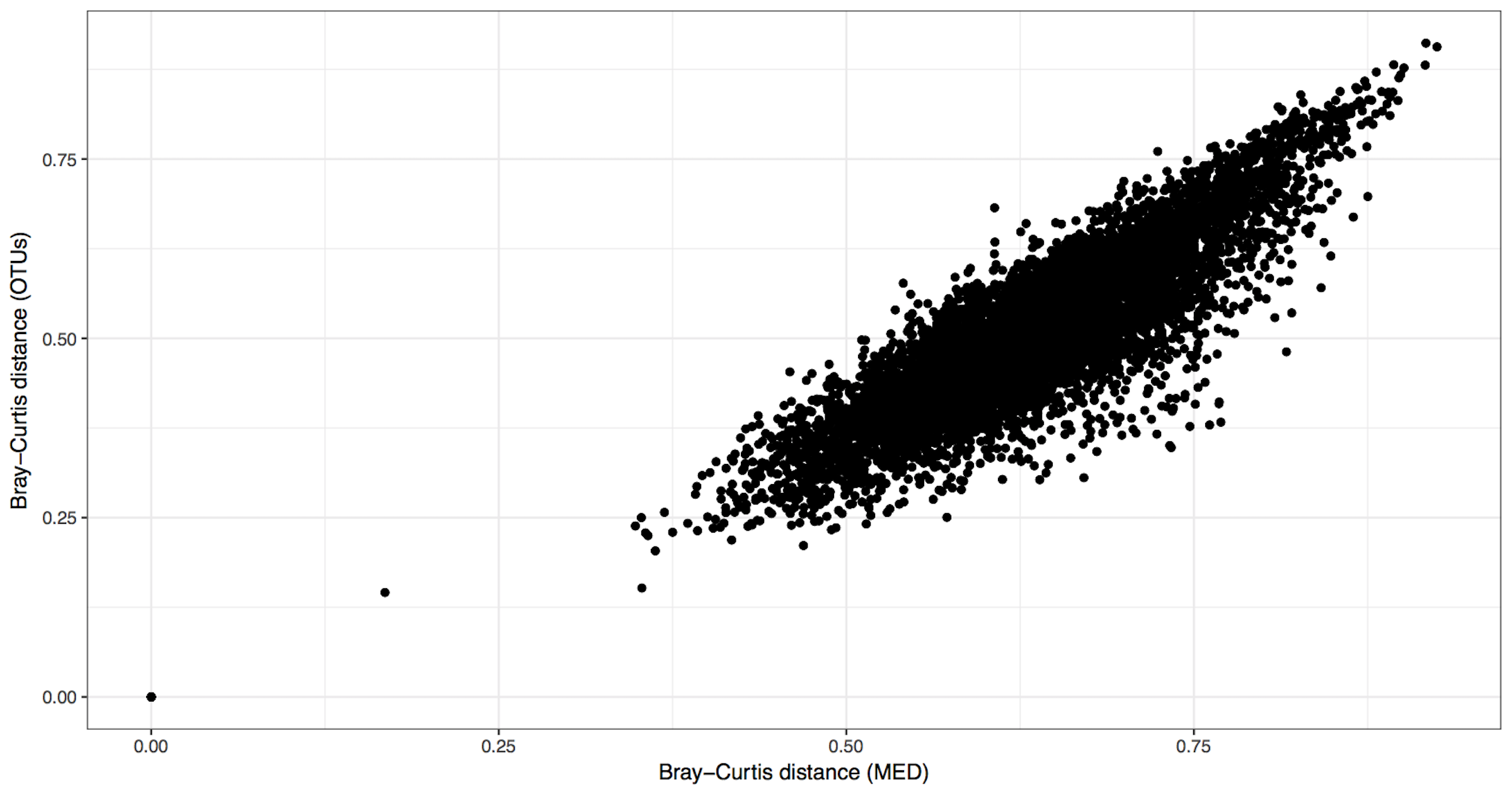

Supplement: FIG S2 [file mbo004173481sf2.tif]

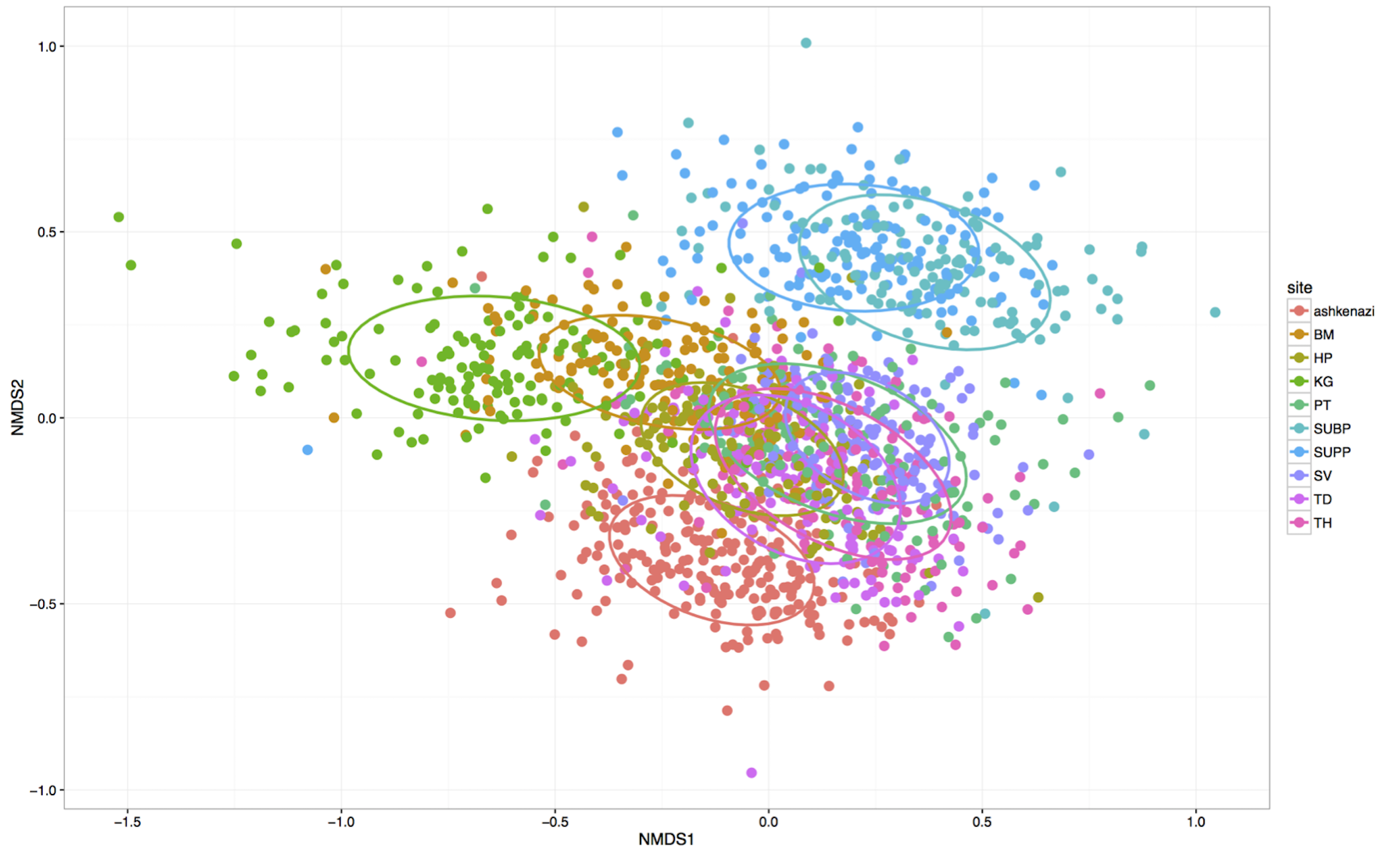

Supplement: FIG S3 [file mbo004173481sf3.tif]

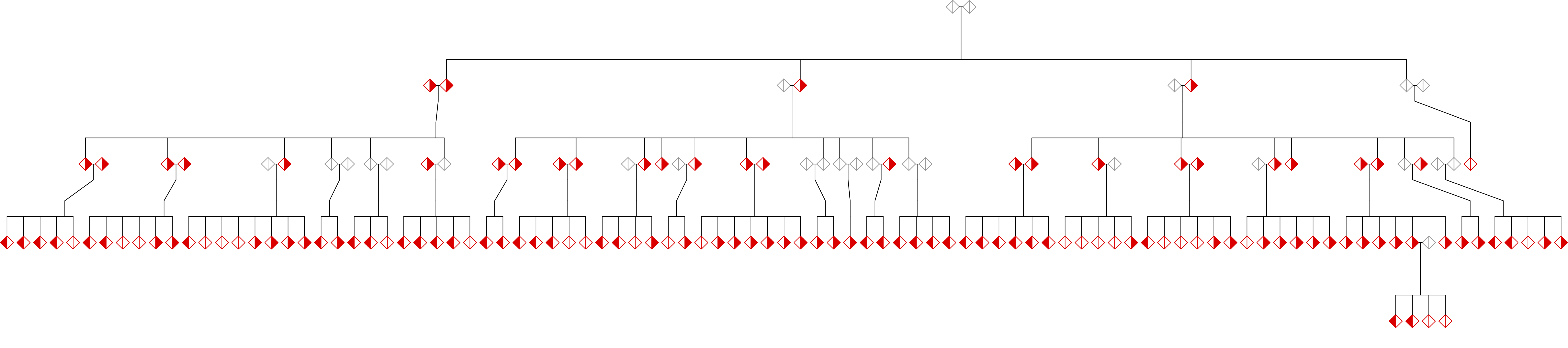

Supplement: FIG S4 [file mbo004173481sf4.tif]

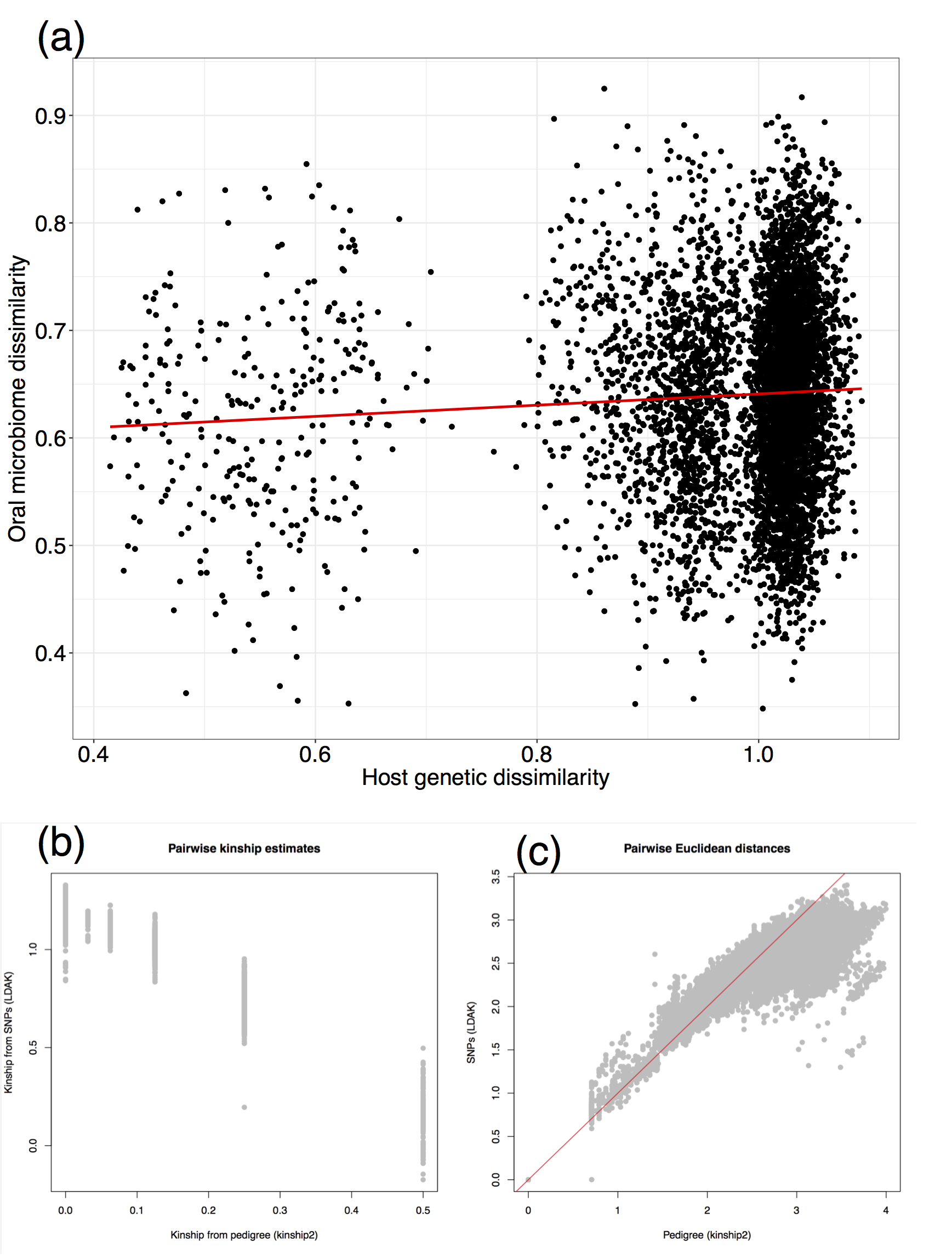

Supplement: FIG S5 [file mbo004173481sf5.tif]

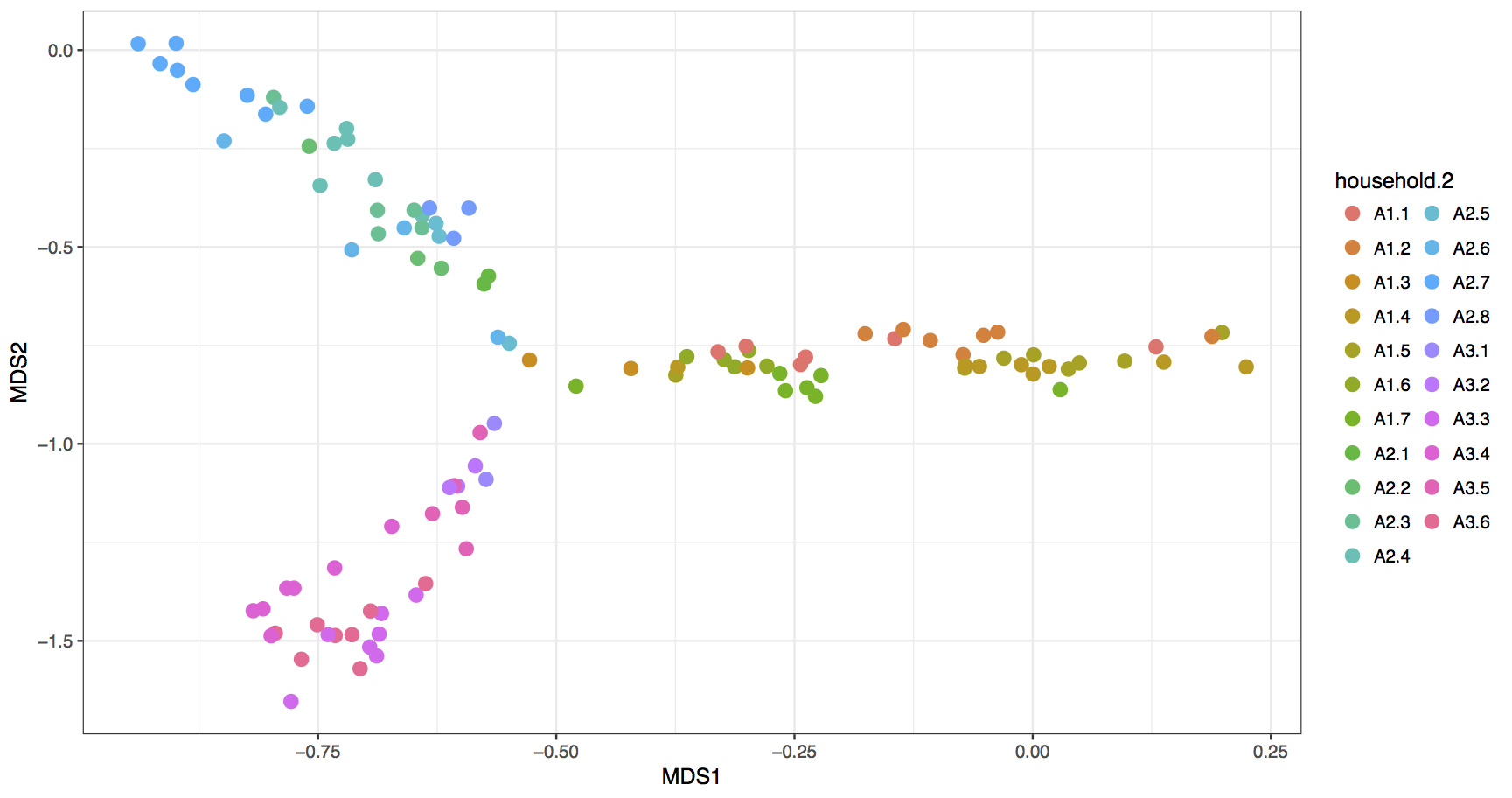

Supplement: FIG S6 [file mbo004173481sf6.tif]

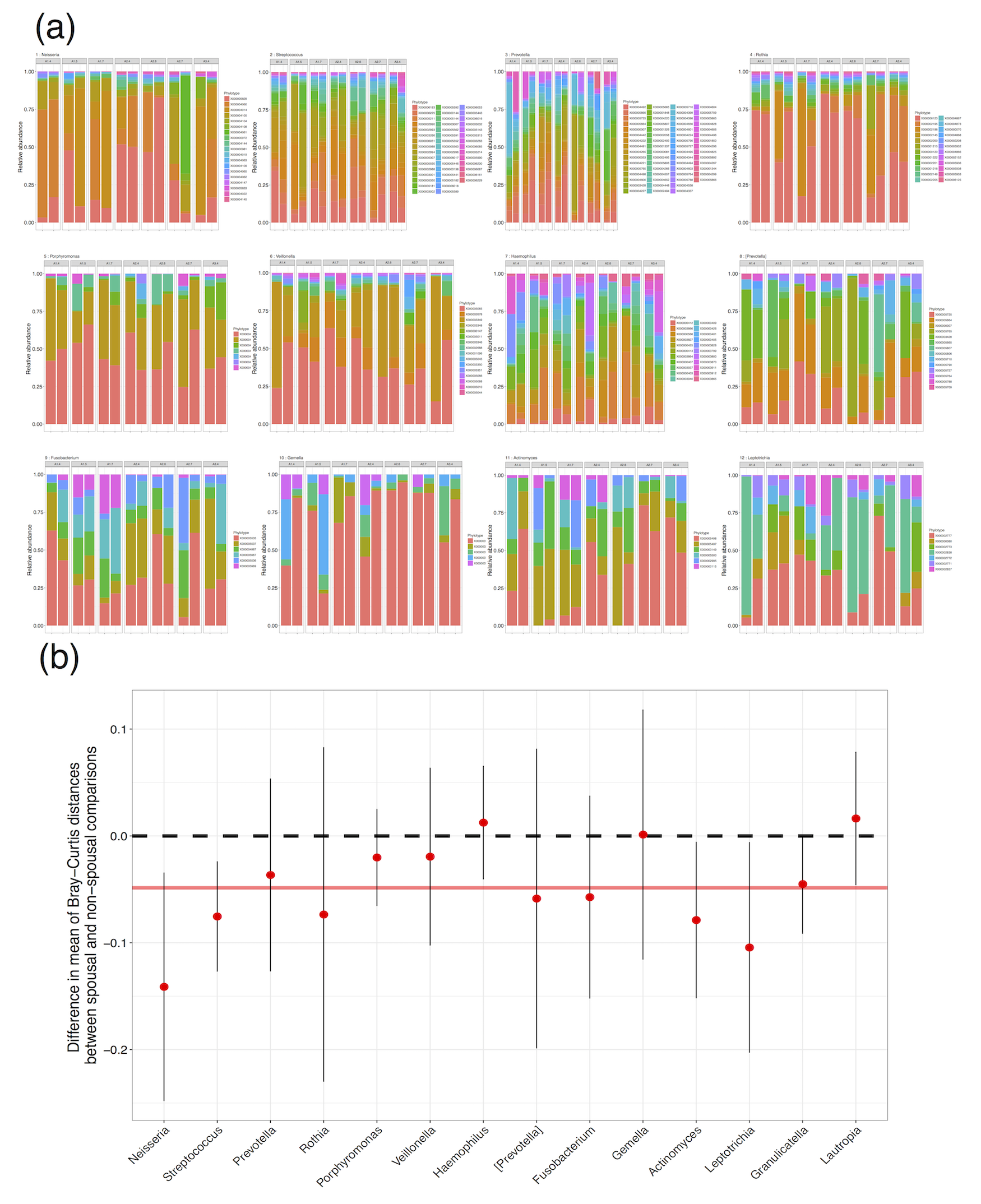

Supplement: FIG S7 [file mbo004173481sf7.tif]
