# Supplementary material for: The Human Salivary Microbiome Is Shaped by Shared Environment Rather than Genetics: Evidence from a Large Family of Closely Related Individuals
Source: mBio. 2017 Sep 12;8(5):e01237-17. doi: 10.1128/mBio.01237-17 (PMC5596345; doi:10.1128/mBio.01237-17)
Supplement: TEXT S1 [file mbo004173481s1.docx]

Supplementary Text 1

This supplementary material file contains the following sections:

1. **Expanded Protocol.** Details of sample collection, DNA extraction, spikes, and sequencing.
2. **Analysis.** Details of subsequent analysis.

**1. Expanded Protocol**

**Sample collection**

Preparation of 2X saliva preservative buffer: 50 mM Tris pH 8.0, 50 mM EDTA pH 8.0, 200 mM NaCl, 1% (w/v) SDS and 50mM sucrose dissolved in ddH_2_O. Filter sterilize through a 0.2 µm filter.

Two ml of saliva was collected from each participant and an equal volume of 2x saliva preservative buffer was added. After that, 15µl of proteinase K (Sigma-Aldrich Company Ltd, Dorset, UK), 75µl of 10% SDS and 2µl of 10% azide per millilitre was added to the samples and incubated overnight at 50°C and stored at -20^0^C.

**DNA extraction**

The PurElute™ Bacterial Genomic Kit (Edge Biosystems, Gaithersburg, MD) was used to extract the genomic DNA according the manufacturer’s instructions. We extracted DNA from 0.5 ml sample/buffer mix and finally re-suspended the dried DNA pellet in 40μl of DNase RNase free water.

**Spikes**

Three constructs were designed to be inserted in all samples for quantification and quality control purposes. The constructs comprised of the following sequences:

Construct 1: gatcga signature

GGATTAGATACCCGGGTAGTCCTCTAGCTAGCTAGTGCAGCGATGCTAGCTAGATGCATGCAGCATGCTGATAGCGTCGATGTAGCTAGCTAT**gatcga**ACGTACGTCCATCTAGCACGTCACGTCAGCTGCAGCGTCGTAATTGCCAATAACGCGCCACACTGTGTGTGTCACACGTGTGGTGCACTACGAGAGTGTGCATCTCTCTTGGTGTTGGCACAATCGACGATATACAGCGGAGCTACTGTATTACACACACCGTAT**gatcga**CAGTCTATTGTGGATGCTACCACTATCTATCTAGCGTACACCTGATCTTACACATTCACGGAGGAAGGCGGGGACGACGT

Construct 2: cagtac signature

GGATTAGATACCCGGGTAGTCCTCTAGCTAGCTAGTGCAGCGATGCTAGCTAGATGCATGCAGCATGCTGATAGCGTCGATGTAGCTAGCTAT**cagtac**ACGTACGTCCATCTAGCACGTCACGTCAGCTGCAGCGTCGTAATTGCCAATAACGCGCCACACTGTGTGTGTCACACGTGTGGTGCACTACGAGAGTGTGCATCTCTCTTGGTGTTGGCACAATCGACGATATACAGCGGAGCTACTGTATTACACACACCGTAT**cagtac**CAGTCTATTGTGGATGCTACCACTATCTATCTAGCGTACACCTGATCTTACACATTCACGGAGGAAGGCGGGGACGACGT

Construct 3: tactag signature

GGATTAGATACCCGGGTAGTCCTCTAGCTAGCTAGTGCAGCGATGCTAGCTAGATGCATGCAGCATGCTGATAGCGTCGATGTAGCTAGCTAT**tactag**ACGTACGTCCATCTAGCACGTCACGTCAGCTGCAGCGTCGTAATTGCCAATAACGCGCCACACTGTGTGTGTCACACGTGTGGTGCACTACGAGAGTGTGCATCTCTCTTGGTGTTGGCACAATCGACGATATACAGCGGAGCTACTGTATTACACACACCGTAT**tactag**CAGTCTATTGTGGATGCTACCACTATCTATCTAGCGTACACCTGATCTTACACATTCACGGAGGAAGGCGGGGACGACGT

These constructs were cloned in the kanamycin resistant *E. coli* vector PJ201 (DNA2.0, Newark, CA). Transformation was carried out using a standard heat-shock protocol (heat shock at 42° C for 40 s), and plated on Luria-Bertani (LB) agar with kanamycin (50 µg/ml). The colonies that developed were subcultured in 5 mL of LB broth with kanamycin (50µg/ml) and incubated overnight. Plasmids were isolated using the QIAprep Spin Miniprep Kit (Qiagen, UK) following the manufacturer’s instructions. The plasmids DNA were sequenced and only those samples with 100% match were used.

A 10X stock solution was prepared in RNase/DNase free water:

- 0.04 ng/ml Construct 1 gatcga signature (working concentration of 0.004 ng/ml)
- 0.004 ng/ml Construct 2 cagtac signature (working concentration of 0.0004 ng/ml)
- 0.0008 ng/ml Construct 3 tactag signature (working concentration of 0.00008 ng/ml)

Two microliters of the stock solution were added to 18μl of each DNA sample and sent for sequencing.

**PCR amplification, purification and sequencing**

The Mastermix 16S Basic (Molzym GmbH & Co.KG, Bremem, Germany) containing MolTaq 16S DNA polymerase was used to generate PCR amplicons. PCR amplicons were purified in two rounds using the Agencourt® AMPure® XP system (Beckman Coulter, Beverly, Massachusetts) in an automated liquid handler Hamilton StarLet (Hamilton Company, Boston, Massachusetts). Two rounds of PCR clean-up were carried out with the following modifications:

1) 50 µL of Agencourt AMPure XP of beads were added to the 50 µL of PCR products (1X).

2) The beads were air dried for 5-10 minutes

3) After drying during the first clean-up, beads were resuspended in 52 µL of 10 mM TRIS-Acetate, pH 8.0 in reagent grade water and 50 µL removed for the second clean-up.

4) Steps 1-2 were repeated for the second clean-up.

5) After drying, beads were resuspended in 32 µL of µL of 10 mM TRIS-Acetate, pH 8.0 in reagent grade water and 30 µL removed for quantification, pooling and sequencing.

6) DNA quantitation and quality control was performed using the Agilent 2100 Bioanalyzer system (Agilent Technologies, Inc., Santa Clara, CA). PCR amplicons showed a peak of an average molecular weight of 500 bp, with very low to no small fractions (~120-140 bp) that could represent primer dimer hybridization.

A set of primers were used to amplify the V5-V7 region of the 16S rRNA gene on the Illumina MiSeq System Illumina, San Diego, CA). PCR was performed using the forward primer 785F (GGATTAGATACCCBRGTAGTC) and the reverse primer 1175R (ACGTCRTCCCCDCCTTCCTC) as described previously (1, 2).

**2. Analysis**

**Comparison of MED against OTUs**

For using MED (1) on 3,360,574 sequences to find 217 phylotypes we used the following default parameters: minimum substantive abundance of an oligotype, M, 627 (1/5000 total sequences); maximum nucleotide variation allowed within an oligotype, d, 4. 1,044,114 sequences were removed as outliers during Minimum Entropy Decomposition due to the minimum substantive abundance criterion (853,159) and the nucleotide variation criterion (190,955). For a table of counts of phylotypes by sample, and taxonomic assignment of phylotypes along with representative sequences, see Supplementary Table 1.

In order to compare MED (3) with OTU picking, we also used VSEARCH v1.11.1 (4) to cluster OTUs de novo at 98.5% sequence similarity, finding 566 OTUs. Comparison of Bray-Curtis distances between samples calculated between methods showed a strong correlation (Spearman’s $\rho=0.88$, *p*<0.001, Supplementary Figure 2). We then conducted a permutational analysis of the variance in Bray-Curtis distances from either MED phylotype or OTU composition, using adonis on 177 samples from Family A, Family B, and unrelated controls (‘Family’) using sequencing plate, family, gender, and age as explanatory variables. Distances computed from MED phylotype composition resulted in a greater F-statistic (ratio of between-group/within-group variance) in all cases, confirming that MED phylotypes allow more explanation of variance as intended (3). All further analysis used MED phylotypes rather than OTUs.

| Variable | OTU *F-*statistic (*p*-value) | MED *F-*statistic (*p*-value) |
| --- | --- | --- |
| Sequencing plate | 2.876 (0.001) | 3.132 (0.001) |
| Family | 2.134 (0.004) | 2.319 (0.001) |
| Gender | 0.963 (0.442) | 1.135 (0.243) |
| Age | 2.516 (0.009) | 2.560 (0.001) |

As this analysis found a significant effect of sequencing plate and age, we included these in all further analyses as covariates.

**Effect of Crohn’s disease status**

28 individuals within the dataset had Crohn’s disease at the time of sampling. However, we found no effect of Crohn’s in a permutational analysis of variance using adonis on *n*=145 individuals, controlling for other variables.

|  | Df | SumsOfSqs | MeanSqs | F.Model | R2 | Pr(>F) |
| --- | --- | --- | --- | --- | --- | --- |
| Sequencing run | 1 | 0.71 | 0.71 | 3.24 | 0.02 | 0.001 |
| Gender | 1 | 0.27 | 0.27 | 1.26 | 0.01 | 0.13 |
| Age | 1 | 0.55 | 0.55 | 2.52 | 0.02 | 0.001 |
| Crohn's | 1 | 0.30 | 0.30 | 1.37 | 0.01 | 0.101 |
| Residuals | 140 | 30.45 | 0.22 | NA | 0.94 | NA |
| Total | 144 | 32.28 | NA | NA | 1.00 | NA |

**Inclusion of host genetics**

As stated in the main text, we calculated pedigree kinships with kinship2 (5) and genetic kinships with LDAK v5.94 (6). These genetic kinships $k_{g}$ are normalized to have a mean of zero, and correspond to genetic similarity between individuals. $k_{g}$correlates with the pedigree kinship $k_{p}$ but there can be substantial spread around the expected values due to the random nature of genetic inheritance (Supplementary Figure 5b), making $k_{g}$ a more accurate measure of true genetic similarity between individuals (7).

These kinships are differently defined so have different values and scalings (Supplementary Figure 5), although they are correlated. We converted kinships to dissimilarities scaled between 0 and 1 with:

$d_{g}=1-\frac{(k_{g}-min(k_{g}))}{\max\left( k_{g} \right)-min(k_{g})}$, $d_{p}=1-{2k}_{p}$.

We then converted these dissimilarities to Euclidean distances using dist() and then used metaMDS() to produce a multidimensional scaling (MDS) ordination (8). Our approach followed Blekhman et al. (9) who investigated host genetic variation and its association with microbiome composition. We used *k=*5 dimensions, but found that using more did not affect our conclusions. Before using the MDS axes as covariates, we normalised them using a Box-Cox transformation, with parameter $\lambda$ calculated from BoxCox.lambda in the forecast package, using the formula:

$$y^{(\lambda)}=(y^{\lambda}-1)/\lambda$$

**Permutation of variable order in adonis**

Adonis explains variance by a sequential sum-of-squares approach (8). We reasoned that the order of variables given in tables in the main text was the appropriate one for our intended purpose of testing for effects of household after controlling for other variables:

> d.adonis ~ plate+Gender+samplingAge+MDS1+MDS2+MDS3+MDS4+MDS5+household

However, it should be noted that this sequential approach means that variable order in adonis with an unbalanced design can lead to different conclusions about their effects. We decided to investigate the effect of permuting variables in the model formula to see if this changed our conclusions. Here we show results for the *n=*61 dataset i.e. individuals who had cohabited at some point with at least one another. We ran adonis (*n*=999 permutations) on 1000 permutations of the variables in the model formula including household as an environmental variable i.e.

> d.adonis ~ paste(sample(plate,Gender,samplingAge,MDS1,MDS2,MDS3,MDS4,MDS5,household)), collapse="+")

498 permutations resulted in variables dropping out of the model due to the unbalanced design i.e. the variable added no additional information, and therefore we did not include results from these for fairness of comparison. 502 permutations gave a full model. We corrected for multiple testing using the Benjamini-Hochberg correction (10). Age and household were always significant (*q*<0.05) in 502/502 models. Sequencing run was significant in 213/502 models. Gender was never significant. Crucially, no MDS axis of genetic variation was significant in any of the 502 models. We therefore felt justified in our conclusion that household was the dominant factor, and not host genetics, as found with our original variable order.

**References**

1. **Zaura E, Brandt BW, Teixeira de Mattos MJ, Buijs MJ, Caspers MP, Rashid MU, Weintraub A, Nord CE, Savell A, Hu Y, Coates AR, Hubank M, Spratt DA, Wilson M, Keijser BJ, Crielaard W**. 2015. Same Exposure but Two Radically Different Responses to Antibiotics: Resilience of the Salivary Microbiome versus Long-Term Microbial Shifts in Feces. MBio. 10;6(6):e01693-15. doi: 10.1128/mBio.01693-15.
2. **Kraneveld EA, Buijs MJ, Bonder MJ, Visser M, Keijser BJF, Crielaard W, Zaura E.** 2012. The Relation between Oral Candida Load and Bacterial Microbiome Profiles in Dutch Older Adults. PLoS One. https://doi.org/10.1371/journal.pone.0042770
3. **Eren AM**, **Morrison HG**, **Lescault PJ**, **Reveillaud J**, **Vineis JH**, **Sogin ML**. 2014. Minimum entropy decomposition: Unsupervised oligotyping for sensitive partitioning of high-throughput marker gene sequences. ISME J **9**:968–979.
4. **Rognes T**, **Flouri T**, **Nichols B**, **Quince C**, **Mahé F**. 2016. VSEARCH: a versatile open source tool for metagenomics. PeerJ **4**:e2584.
5. **Sinnwell JP**, **Therneau TM**, **Schaid DJ**. 2014. The kinship2 R package for pedigree data. Hum Hered **78**:91–3.
6. **Speed D**, **Hemani G**, **Johnson MR**, **Balding DJ**. 2012. Improved heritability estimation from genome-wide SNPs. Am J Hum Genet **91**:1011–21.
7. **Speed D**, **Balding DJ**. 2014. Relatedness in the post-genomic era: is it still useful? Nat Rev Genet **16**:33–44.
8. **Oksanen J**. 2016. vegan: Community Ecology Package.
9. **Blekhman R**, **Goodrich JK**, **Huang K**, **Sun Q**, **Bukowski R**, **Bell JT**, **Spector TD**, **Keinan A**, **Ley RE**, **Gevers D**, **Clark AG**. 2015. Host genetic variation impacts microbiome composition across human body sites. Genome Biol **16**:191.
10. **Benjamini Y**, **Hochberg Y**. 1995. Controlling the False Discovery Rate: A Practical and Powerful Approach to Multiple Testing. J R Stat Soc Ser B **57**:289–300.
